# Hematological profile of blood parasitic infected dogs in Southern Thailand

**DOI:** 10.14202/vetworld.2020.2388-2394

**Published:** 2020-11-10

**Authors:** Sorawat Thongsahuan, Usa Chethanond, Siriwat Wasiksiri, Vannarat Saechan, Wichaya Thongtako, Tipayaratn Musikacharoen

**Affiliations:** Faculty of Veterinary Science, Prince of Songkla University, Songkhla, Thailand

**Keywords:** blood parasite, dogs, hematological characteristics, Southern Thailand

## Abstract

**Background and Aim:**

Tick-borne pathogens such as *Babesia canis*, *Hepatozoon canis*, and *Ehrlichia canis* can cause serious disease in canines. Each blood parasite can be associated with different hematological characteristics in infected dogs. Identification of hematological alterations during routine laboratory screening of blood samples from dogs displaying clinical signs is essential for diagnosing blood parasitic infections. This study aimed to evaluate parasitic infections and hematological alterations in blood samples of infected dogs in Southern Thailand.

**Materials and Methods:**

A total of 474 blood samples were collected from dogs presented at the Veterinary Teaching Hospital of the Prince of Songkla University between 2016 and 2019. An automatic hematology analyzer was used to establish hematological values; peripheral blood films were screened for blood parasites and their detection was associated with hematological alterations to determine the odds ratio (OR).

**Results:**

This study found that *E. canis* (n=127) was the most common blood parasite infecting dogs in southern Thailand, followed by *H. canis* (n=100) and *B. canis* (n=24). Hematological alterations caused by *Ehrlichia* infections included anemia, thrombocytopenia, monocytosis, and eosinophilia (OR=14.64, 17.63, 20.34, and 13.43, respectively; p<0.01). The blood samples of *Hepatozoon*-infected dogs were characterized by anemia, thrombocytopenia, leukocytosis, neutrophilia, and monocytosis (OR=6.35, 3.16, 12.80, 11.11, and 17.37, respectively; p<0.01). Anemia, thrombocytopenia, eosinopenia, and lymphopenia (OR=10.09, 33.00, 20.02, and 66.47 respectively; p<0.01) were associated with *B. canis*-infected dogs.

**Conclusion:**

These data support the fact that hematological abnormalities are a hallmark for the identification of tick-borne infections. The hematological values, hereby reported, can be used as a guideline for the clinical diagnosis of canine blood parasitic infections in Southern Thailand.

## Introduction

Tick-borne pathogens, including protozoans, bacteria and viruses, can cause serious illnesses in both humans and domestic animals, particularly in dogs. The main canine tick-borne diseases; babesiosis, hepatozoonosis, and ehrlichiosis are transmitted by the important hard tick vector, *Rhipicephalus sanguineus*, commonly called the brown dog tick [1]. Dogs infected by different tick-borne pathogens typically present with similar clinical signs such as high fever, drowsiness, loss of appetite, pale mucous membranes, vomiting, and weight loss [2]. Babesiosis is caused by the intraerythrocytic protozoa, *Babesia* spp., which is transmitted when ticks bite and release protozoal sporozoites from their salivary glands into a dog’s blood stream. The two important parasites causing canine disease can be differentiated based on their morphologically distinct forms in the erythrocytes of infected hosts: The organism of *Babesia canis* and *B. gibsoni* is large pear-shaped and small round to oval, respectively [3]. Infection typically results into red blood cell destruction and subsequent anemia [1]. In Thailand, most canine babesiosis cases are caused by *B. canis* [4-6]; nonetheless, a single study reported the detection of *B. gibsoni* antigen by serological test [7]. Canine hepatozoonosis is caused by the protozoal parasite, *Hepatozoon canis*, which infects neutrophils, resulting into a decreased immune response in infected dogs. Unlike most other hematopoietic diseases, canine hepatozoonosis is transmitted to new hosts after ingestion of ticks containing sporozoites [8]. *H. canis* is commonly detected in Thai dogs, including both sick and healthy animals [6,9,10]. In contrast, canine ehrlichiosis or tropical canine pancytopenia is caused by the tick-transmitted intracellular bacterium, *Ehrlichia* spp. The bacterial agglomerates form morulae within the host’s monocytes after being transmitted through a tick bite [1]. *Ehrlichia canis* is a common species reported in Thailand [6,11-13].

The diagnosis of tick-borne diseases is performed usually based on the observation of clinical signs in conjunction with laboratory testing. Microscopic examination of blood smears is the conventional and routine diagnostic method, as it allows the identification of blood parasites based on their morphology. This technique is not expensive and detects acute infections successfully; however, it requires skilled personnel, is time-consuming, and has low sensitivity. Serological tests also are used frequently, but cross-reactions have been reported while the current infection status cannot be determined [14-16]. Polymerase chain reaction (PCR) assays have been developed to diagnose blood parasites, and they yielded high sensitivity and specificity [6,11,13,17]. Nonetheless, molecular testing necessitates special equipment and is relatively expensive when compared to microscopic or serological methods. All tick-borne infectious diseases can affect hematological characteristics and induce changes directly or indirectly [10,18-21]. Screening hematological alterations is, therefore, very important in routine laboratory testing of blood parasites, including malarial diagnosis in humans [22-24]. Thongsahuan *et al*. [10] previously reported the prevalence of blood parasites and hematological changes associated with low *H. canis* parasitemia in healthy dogs from Songkhla Province (Southern Thailand), which differed from other regions [9,11-13,18].

Due to the limited availability of hematological data associated with canine hepatozoonosis, babesiosis, and ehrlichiosis in Southern Thailand, this study aimed to compare the hematological profiles between infected and healthy dogs from this region to identify hematological alterations caused by different blood parasites, and correlate them with the pathogenesis of these diseases.

## Materials and Methods

### Ethical approval

All animal procedures were performed by veterinarians, and ethical approval was obtained from the Institutional Animal Care and Use Committee, the Prince of Songkla University (Ref. 31/55).

### Blood samples

This study was conducted on 474 dogs collected from several provinces in Southern Thailand, and presented at the Veterinary Teaching Hospital of the Prince of Songkla University between 2016 and 2019. Approximately 3 mL of blood were collected by venipuncture and placed into heparin tubes. The dogs included in this study, were assigned to two groups: Healthy dogs over 1-year-old with no known disease (n=223) and infected dogs diagnosed with tick-borne disease (n=251).

### Laboratory investigation

Complete blood count (CBC) was performed using a Mindray BC-5000 Vet auto hematology analyzer (Mindray, Shenzhen, China) [25]. Hematological profiles consisting of red blood cell (RBC) count, hemoglobin (HGB), hematocrit (HCT), mean corpuscular volume (MCV), mean corpuscular hemoglobin (MCH), MCH concentration (MCHC), red blood cell distribution width (RDW), white blood cell (WBC) count, platelet, and WBC differential count were recorded and analyzed. Thin blood films were prepared from each blood sample and stained with 10% Giemsa to screen for blood parasites using a light microscope at high magnification (400× and 1000×) (Nikon, Japan).

### Statistical analysis

Descriptive statistics (mean and standard deviations) were used to analyze the data. The independent sample t-test was conducted to compare mean scores of CBC parameters between the two groups using Microsoft Excel. Correlations between hematological profiles and each blood parasite were performed by the Chi-square test and odds ratio (OR) with a 95% confidence interval obtained using the R program. All p<0.05 were considered as statistically significant.

## Results

**Figure-1:**
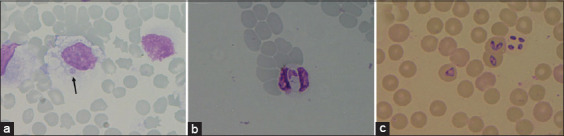
Canine blood smear showing (a) *Ehrlichia canis* in monocyte (arrow) (b) *Hepatozoon canis* in neutrophil and (c) *Babesia canis* in red blood cell (1000**×**).

**Figure-2:**
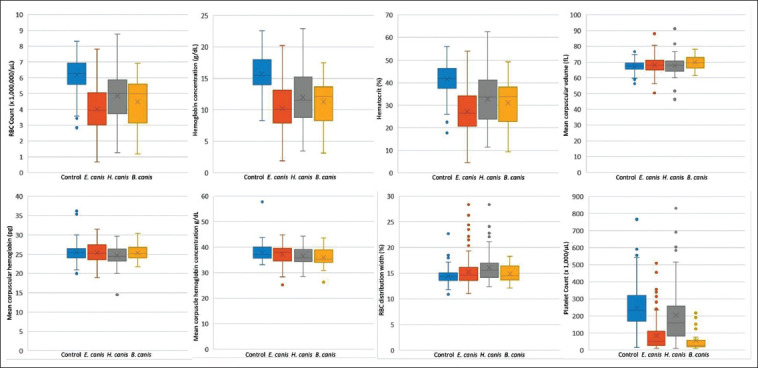
Box-and-Whisker Plots representing variation in red blood cell parameters and platelet of blood samples from healthy dogs (control) and various tick-borne infected dogs.

**Figure-3:**
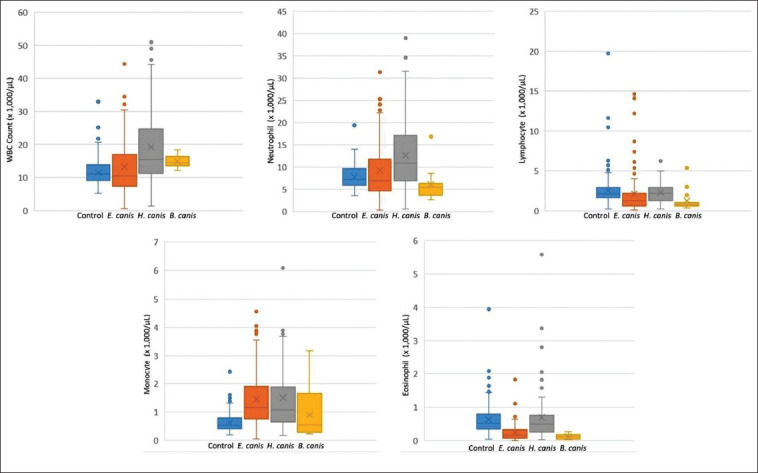
Box-and-Whisker plots representing variation in white blood cell parameters of blood samples from healthy dogs (control) and various tick-borne infected dogs.

Of the 251 blood samples collected from dogs infected by tick-borne parasites, 127 were positive for *E. canis* (50.60%), 100 for *H. canis* (39.84%), and 24 for *B. canis* (9.56%, Figure-1). No blood parasitic coinfection was detected. All blood samples collected from healthy dogs (n=223) were negative for tick-borne parasites. The average hematological values obtained from the healthy and infected groups, as well as a summary of interpreted data, are presented in Tables-1 and 2 [26]. A significant difference in the hematological parameters of canine blood samples in healthy and various parasitic infected groups are displayed in Box-and-Whisker Plots (Figures-2 and 3).

**Table-1:** Mean values of hematological profiles of dogs infected with *Ehrlichia canis, Hepatozoon canis*, and *Babesia canis* compared to healthy dogs from Southern Thailand.

Parameters	Units	Reference ranges [26]	Healthy dogs (n=223)	*Ehrlichia canis (n=127)*	*Hepatozoon(* *canis* n=100)	*Babesia* *canis (n=24)*
RBC	10^6^cells/µL	5.5-8.5	6.17±1.00	4.02±1.51^a^	4.84±1.57^a^	4.49±1.45^a^
Hemoglobin	g/dL	12-19	15.70±2.77	10.23±4.07^a^	12.01±4.26^a^	11.33±3.65^a^
Hematocrit	%	37-57	41.51±6.71	27.18±10.08^a^	32.78±11.06^a^	31.08±9.80^a^
MCV	fL	66-77	67.30±3.36	68.10±5.02	67.70±5.33	69.92±4.32^a^
MCH	pg	19.5-24.5	25.49±2.11	25.40±2.52	24.70±2.26^a^	25.37±2.12
MCHC	%	32-36	37.89±2.94	37.24±3.65	36.50±3.28^a^	35.99±3.75^b^
RDW	%	12-15	14.42±1.34	15.28±2.69^a^	16.06±2.65^a^	14.87±1.88
WBC	10^3^ cells/µL	6-17	11.57±3.70	13.22±8.37^b^	19.24±11.12^a^	9.72±6.09
Neutrophil	10^3^ cells/µL	3-11.5	7.72±2.53	9.25±7.02^b^	12.58±7.28^a^	6.01±3.37
Lymphocyte	10^3^ cells/µL	1-4.8	2.55±1.90	2.14±2.69	2.30±1.40	1.27±1.28^b^
Monocyte	10^3^ cells/µL	0.15-1.35	0.61±0.32	1.44±0.98^a^	1.50±1.18^a^	0.89±0.85
Eosinophil	10^3^ cells/µL	0.1-1.25	0.62±0.44	0.23±0.24^a^	0.69±0.83	0.11±0.07^a^
Platelet	10^3^ cells/µL	200-500	246.27±121.99	83.67±87.12^a^	202.54±162.14^b^	54.70±57.30^a^

a Significant difference at p<0.01,

b Significant difference at p<0.05. WBC=White blood cell, RBC=Red blood cell, MCV=Mean corpuscular volume, MCH=Mean corpuscular hemoglobin, MCHC=Mean corpuscular hemoglobin concentration, RDW=Red blood cell distribution width

**Table-2:** Interpretation of hematological profiles of dogs infected with *Ehrlichia canis, Hepatozoon canis*, and *Babesia canis.*

Parameters	Percentage of dogs with values outside reference ranges								

	*Ehrlichia canis*		Results	*Hepatozoon canis*		Results	*Babesia canis*		Results
		
	Lower (%)	Higher (%)		Lower (%)	Higher (%)		Lower (%)	Higher (%)	
Hematocrit	81.1	0	Anemia	63.0	3.0	Anemia	75	0	Anemia
WBC	14.2	23.6	Leukocytosis	2.0	44.0	Leukocytosis	26.1	17.3	Leukopenia
Neutrophil	8.2	27.5	Neutrophilia	2.7	48.0	Neutrophilia	7.1	7.1	-
Lymphocyte	40.3	11.9	Lymphopenia	21.3	6.7	Lymphopenia	73.3	6.7	Lymphopenia
Monocyte	2.7	41.2	Monocytosis	0	38.7	Monocytosis	0	26.7	Monocytosis
Eosinophil	30.3	0.9	Eosinopenia	5.3	13.3	Eosinophilia	40.0	0	Eosinopenia
Platelet	90.6	0.8	Thrombocytopenia	61.0	6.0	Thrombocytopenia	95.6	0	Thrombocytopenia

Correlations between hematological factors and each detected blood parasite are shown in Table-3. The RBC counts, HGB, and HCT of all infected dogs were significantly lower when compared to that in the healthy group, resulting in anemia (HCT *E. canis* OR=14.64, *H. canis* OR=6.35, and *B. canis* OR=10.09; p<0.01, Table-3). The MCV of *B. canis*-infected dogs and MCH of *H. canis*-infected dogs were significantly higher (OR=0.68) and lower (OR=0.43), respectively, than that in the healthy group (p<0.01, Table-3). In addition, both *H. canis*- and *B. canis*-infected dogs showed significantly reduced MCHC values (OR=0.48 and 0.32, respectively, Table-3). It was interesting that thrombocytopenia correlated positively with all blood parasitic infections (platelet *E. canis* OR=17.63, *H. canis* OR=3.16, and *B. canis* OR=33.00, respectively, Table-3).

**Table-3:** Correlations between hematological profiles and canine blood parasitic infections (*Ehrlichia canis, Hepatozoon canis*, and *Babesia canis*).

Parameters	Factors	OR (95% CI)		

		*Ehrlichia canis* infection	*Hepatozoon canis* infection	*Babesia canis* infection
RBC	<5.5×10^6^ cells/µL	15.19 (8.83-27.19)^a^	5.79 (3.48-9.79)^a^	7.25 (2.94-19.90)^a^
Hemoglobin	<12 g/dL	19.97 (11.24-36.89)^a^	9.46 (5.25-17.59)^a^	5.72 (2.20-14.56)^a^
	>19 g/dL	1.04 (0.28-3.00)	1.05 (0.33-2.81)	-
Hematocrit	<37%	14.64 (8.61-25.74)^a^	6.35 (3.79-10.83)^a^	10.09 (3.96-29.57)^a^
MCV	<66 fL	1.19 (0.74-1.91)	1.38 (0.83-2.28)	0.68 (0.21-1.79)
MCH	>24.5 pg	0.65 (0.42-1.04)	0.43 (0.26-0.70)^a^	0.62 (0.26-1.53)
MCHC	>36%	1.01 (0.62-1.67)	0.48 (0.29-0.78)^a^	0.32 (0.12-0.78)^a^
RDW	<12%	2.17 (0.22-21.16)	-	-
	>15%	1.91 (1.20-3.05)	3.51 (2.14-5.81)^a^	1.98 (0.81-4.72)
WBC	<6×10^3^ cells/µL	22.00 (6.10-152.80)^a^	3.83 (0.39-37.45)	43.73 (8.78-355.16)^a^
	>17×10^3^ cells/µL	6.00 (3.03-12.52)^a^	12.80 (6.59-26.45)^a^	4.96 (1.22-16.76)^a^
Neutrophil	>11.5×10^3^ cells/µL	4.93 (2.50-10.18)^a^	11.11 (5.54-23.44)^a^	1.08 (0.04-6.29)
Lymphocyte	<0.15×10^3^ cells/µL	16.57 (7.67-40.43)^a^	5.85 (2.42-15.34)^a^	66.47 (16.90-358.98)^a^
	>1.35×10^3^ cells/µL	4.95 (1.95-13.32)^a^	1.88 (0.53-6.00)	7.05 (0.23-68.88)
Monocyte	>1.35×10^3^ cells/µL	20.34 (8.83-56.07)^a^	17.37 (7.20-49.36)^a^	10.17 (2.22-42.62)^a^
Eosinophil	<0.1×10^3^ cells/µL	13.43 (5.45-41.17)^a^	2.08 (0.48-8.41)	20.02 (5.01-85.47)^a^
	>1.25×10^3^ cells/µL	0.17 (0.01-0.88)^b^	1.85 (0.75-4.40)	-
Platelet	<200×10^3^ cells/µL	17.63 (9.29-36.69)^a^	3.16 (1.92-5.30)^a^	33.00 (6.75-794.74)^a^
	>500×10^3^ cells/µL	1.71 (0.06-11.00)	0.58 (0.02-3.39)	-

OR (95% CI)=Odds ratio with a 95% confidence interval,

a significant difference at p<0.01,

b significant difference at p<0.05, WBC=White blood cell, RBC=Red blood cell, MCV=Mean corpuscular volume, MCH=Mean Corpuscular Hemoglobin, MCHC=Mean corpuscular hemoglobin concentration

Leukocytosis was associated with *E. canis* (OR=6.00, p<0.05) and *H. canis* (OR=12.80, p<0.01) cases (Table-3). The WBC differential counts revealed that the number of neutrophils and monocytes was significantly higher in *E. canis* (OR=4.93, p<0.05 and OR=20.34, p<0.01) and *H. canis* (OR=11.11 and OR=17.37, p<0.01) cases when compared to that in the healthy group (Table-3). It was noticeable that *B. canis* infections were characterized by significantly lower lymphocyte (OR = 66.47, p<0.05) and eosinophil (OR=20.02, p<0.01) counts, while eosinopenia also was detected from *E. canis*-positive blood samples (OR=13.43, p<0.01; Table-3).

## Discussion

Canine babesiosis, hepatozoonosis, and ehrlichiosis are important tick-borne diseases that infect dogs worldwide [1]. Microscopic examination of blood films associated with hematological profiling is performed routinely by most Thai Veterinary Hospitals to diagnose blood parasitic infections. By this approach, this study found that *E. canis* was the most common blood parasite infecting dogs in Southern Thailand (50.60%, n=127), followed by *H. canis* (39.84%, n=100) and *B. canis* (9.56%, n=24). This study also reported hematological profiles for each detected organism, which can be used as a guideline for the clinical diagnosis of these canine blood parasitic infections in Southern Thailand.

The results of this study indicated that *E. canis*-infected dogs were at higher risk of showing low RBC, HGB, and HCT volumes by 15.19, 19.97, and 14.64 times, respectively. Moreover, no significant difference was found in MCV, MCH, or MCHC values. The results from RBC parameters suggested normocytic normochromic anemia, which is non-regenerative due to bone marrow dysfunction [27]. In fact, a previous study reported that ehrlichiosis was associated with irreversible bone marrow destruction [28]. In addition, *E. canis* infection may lead to anemia as a result of antibody production against erythrocytes, in combination with immune-mediated hemolytic anemia (IMHA) [27]. RBC indices, hereby obtained, were consistent with those previously reported [11,12,18,29]. Furthermore, monocytosis (41.2%) was the main WBC abnormality in dogs with ehrlichiosis, followed by eosinopenia (30.3%) and neutrophilia (27.5%), which was similar to findings from other reports [12,18,20,30,31]. Leukocytosis was hereby observed controversially in 23.6% of *E. canis*-infected dogs, while other studies reported low numbers of WBC [30,31] or no significant difference in WBC counts when compared to healthy dogs [12,19]. In terms of monocytosis, results of this study suggested that *E. canis*-infected dogs were at risk of showing increased monocyte counts by 20.34 times, which was a higher risk than in the previous reports [12]. The platelet counts in *E. canis* cases were 17.63 times lower than in healthy animals, which was indicative of thrombocytopenia. This condition has been associated frequently with *E. canis* infections [12,18,20,30,32,33], even when a single study reported a normal platelet profile in naturally infected dogs with *E. canis* in Northeastern Thailand [11]. Thrombocytopenia is caused by increased platelet consumption during the acute phase of infection, as a result of inflammatory mechanism [3].

Hepatozoonosis was associated with anemia in this study, and RBC indices (RBC count, HGB, HCT, MCH, and MCHC) were below the normal reference ranges in infected dogs, when compared to healthy animals, which is characteristic of normocytic anemia. Anemia is a common finding in canine hepatozoonosis cases, which occasionally can be severe [8,12,18,34,35]. In contrast, WBC counts were increased in the majority of *H. canis*-infected dogs (44%), when compared to healthy animals, which is indicative of leukocytosis. Even if a single study reported different results [34], the high WBC counts hereby observed corresponded to increased neutrophil (48%) and monocyte (37%) numbers, which is consistent with the previous findings [18,36]. This study provided evidence that WBC and neutrophil numbers were 12.80 and 11.11 times more important, respectively, in dogs diagnosed with hepatozoonosis, when compared to reference values. These elevated cell numbers were higher than those observed in other canine blood parasitic infections, which may be due to the inflammatory response induced by tissue invasion and multiplication of *Hepatozoon* organisms.

In most canine babesiosis cases diagnosed in this study, infected dogs presented with regenerative anemia, as demonstrated by lower RBC, HGB, and HCT volumes when compared to reference ranges. Macrocytic anemia (high MCV), hypochromasia (low MCHC), and heterogeneous cell volume (high RDW) also were associated with *B. canis* infections. A reduction in MCHC reflects a normal HGB content in a larger than normal cell [27], which is most likely the direct consequence of parasitizing *Babesia* organisms and damaging RBCs. Thrombocytopenia also was a predominant characteristic of *B. canis*-positive cases, with the majority of infected dogs showing reduced platelet counts (95.6%), which were 33 times lower than the lowest reference range value [37]. Mild leucopenia and neutropenia also were detected, but hematological parameters did not differ significantly from those observed from healthy dogs, while eosinopenia (40%) and lymphopenia (73.3%) were associated significantly with canine babesiosis, as previously found by other studies [12,18].

These data support the fact that hematological abnormalities are a hallmark for the identification of tick-borne infections. The hematological values reported herein can guide veterinarians in clinical diagnosis of canine blood parasitic infections in Southern Thailand. It should be noted that the hematological profiles obtained in this study for canine tick-borne infections were different from those previously published for other Thai regions.

## Conclusion

This study highlights the difference in canine tick-borne infections that can be associated with specific hematological alterations. Canine ehrlichiosis cases presented with anemia, thrombocytopenia, monocytosis, and eosinophilia. Hepatozoonosis infections were characterized by anemia, thrombocytopenia, leukocytosis, neutrophilia, and monocytosis. In contrast, anemia, thrombocytopenia, eosinopenia, and lymphopenia were blood abnormalities of canine babesiosis. In addition, this study demonstrated that dogs showing lower RBC, HGB, HCT, and platelet values than the normal reference ranges are at higher risk of blood parasitic infections when compared to animals with normal hematological profiles.

## Authors’ Contributions

ST conducted the research project, designed the experiments, performed the examinations, analyzed the data, and wrote and edited the manuscript. UC, SW, VS, and WT collected blood samples, performed the examinations, and analyzed the data. TM provided guidance during the entire experiment. All authors read and approved the final manuscript.
